# Exploiting Peptide Self-Assembly for the Development of Minimalistic Viral Mimetics

**DOI:** 10.3389/fchem.2021.723473

**Published:** 2021-07-28

**Authors:** Patrizia Janković, Iva Šantek, Ana Sofia Pina, Daniela Kalafatovic

**Affiliations:** ^1^ Department of Biotechnology, University of Rijeka, Rijeka, Croatia; ^2^ Associate Laboratory i4HB - Institute for Health and Bioeconomy, NOVA School of Science and Technology, NOVA University Lisbon, Caparica, Portugal; ^3^ UCIBIO – Applied Molecular Biosciences Unit, Department of Chemistry, NOVA School of Science and Technology, NOVA University Lisbon, Caparica, Portugal

**Keywords:** self-assembly, viral mimetics, peptides, minimalistic, co-assembly

## Abstract

Viruses are natural supramolecular nanostructures that form spontaneously by molecular self-assembly of complex biomolecules. Peptide self-assembly is a versatile tool that allows mimicking viruses by creating their simplified versions through the design of functional, supramolecular materials with modularity, tunability, and responsiveness to chemical and physical stimuli. The main challenge in the design and fabrication of peptide materials is related to the precise control between the peptide sequence and its resulting supramolecular morphology. We provide an overview of existing sequence patterns employed for the development of spherical and fibrillar peptide assemblies that can act as viral mimetics, offering the opportunity to tackle the challenges of viral infections.

## Introduction

Designed bio-nanomaterials are often inspired by basic processes found in nature such as molecular recognition and self-assembly (Lehn, 2002; Whitesides and Grzybowski, 2002; Yang et al., 2020a). Viruses present a great source of inspiration for the design of life-like materials (Whitesides, 2015; Maslanka Figueroa et al., 2021) as they constitute simple, yet sophisticated supramolecular assemblies that contain genetic code and present well-defined rod-like or spherical morphologies. In addition, they show the ability to self-replicate, respond to physical and chemical stimuli, adapt to the environment, and evade the immune system which makes them ideal candidates to be manipulated and repurposed.

A variety of virus-mimetic materials have been developed for biological and chemical sensing (Mao et al., 2009), drug delivery (Li et al., 2016), cancer immunotherapy (Mohsen et al., 2020) and vaccine design (Abudula et al., 2020). Virus-like particles (VLPs), formed by the multimeric self-assembly of expressed viral structural proteins in absence of genetic material, are the most studied ones (Ludwig and Wagner, 2007; Ferreira and Martins, 2017; Roldão et al., 2019). The complexity of their fabrication, that requires fully folded proteins and efficient upstream and downstream strategies, impacts the production yields, and is associated to high costs. Other examples include polymer peptide nanogels (Lee et al., 2008), dendritic lipopeptides (Liang et al., 2019), iron oxide-lactoferrin magneto-responsive nanocapsules (Fang et al., 2015), peptide-DNA condensates (Cao et al., 2018), rabies-inspired gold nanorods (Lee et al., 2017) or metal–organic frameworks (Qiao et al., 2020). However, the potential of minimalistic, purely peptidic, supramolecular nanostructures to resemble the morphology and/or functionality of viruses has not been fully exploited yet.

Several peptide-based therapeutics have reached the market while others are in various phases of clinical development for the treatment of cancer and metabolic disorders (Vlieghe et al., 2010; Craik et al., 2013). Compared to their protein counterparts, peptides are easier to synthesize and more stable under harsh conditions (Fosgerau and Hoffmann, 2015). Furthermore, peptides can be exploited as building blocks for the fabrication of highly ordered nanostructures with varying morphologies and surface functionalities, developed for drug delivery, tissue engineering, and regenerative medicine due to their inherent biocompatibility and biodegradability (Zhang, 2003; Collier et al., 2010; Woolfson and Mahmoud, 2010; Frederix et al., 2015; Smith et al., 2015; Slocik and Naik, 2017; Lampel et al., 2018; Sharma et al., 2021).

Peptide-based nanomaterials offer simple and low costs alternatives to VLPs (Matsuura, 2012; Hendricks et al., 2017; Singh et al., 2017; Cai et al., 2020). When designing peptide-based virus mimetics, the main strategy is capsid reconstruction through the formation of supramolecular assemblies based on peptide segments with the goal of mimicking the viral architecture and functionality of efficient cell entry, immune evasion, and targeted cargo delivery. In here, we provide an overview of sequence patterns that drive peptide self-assembly, followed by the potential to achieve dimensional control through co-assembly. Finally, examples of peptide-based building blocks used in the design of supramolecular virus mimetics are discussed.

## Morphological Control Through Sequence Design

**FIGURE 1:**
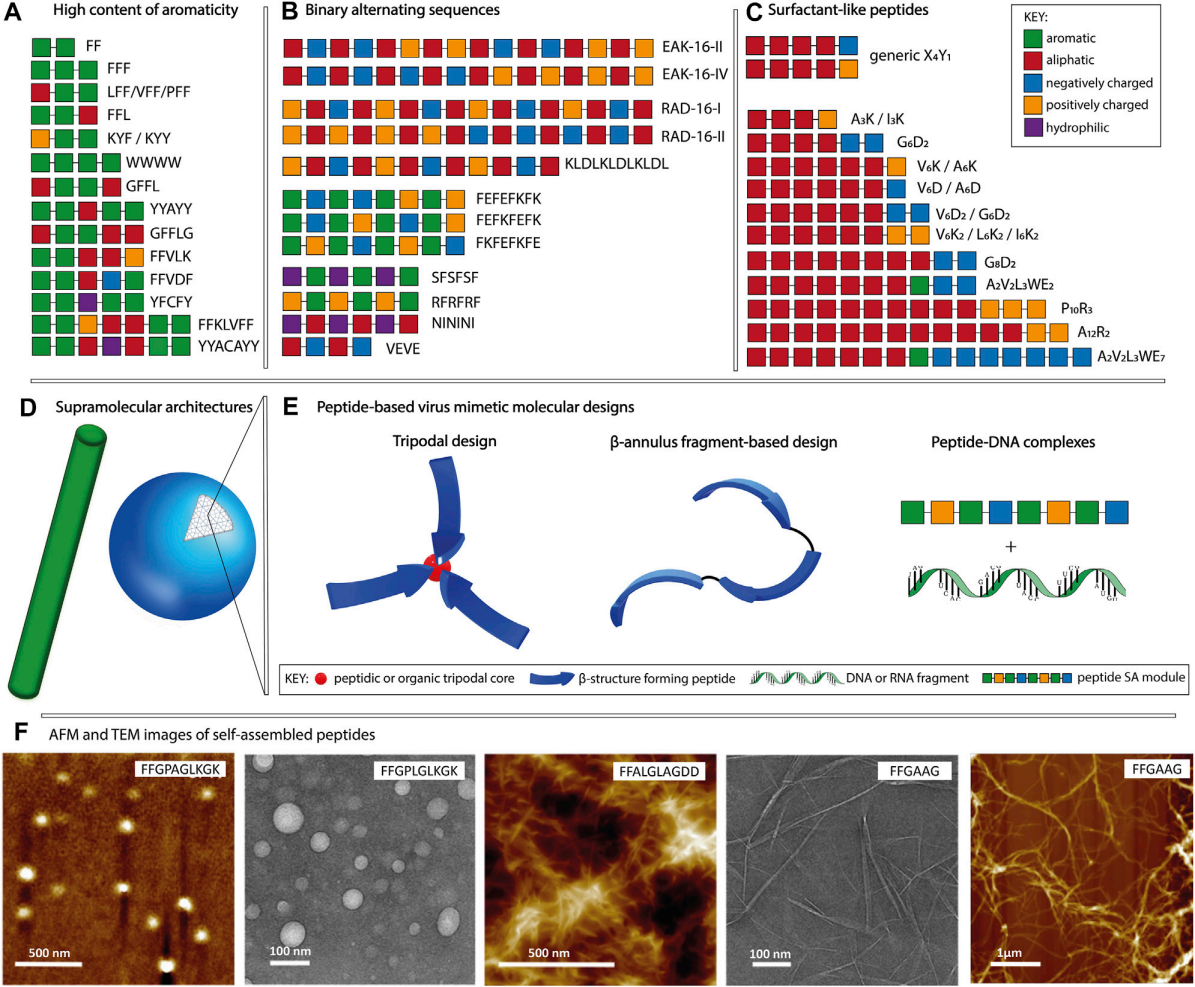
Schematic representation of the main sequence patterns found in self-assembling peptides, namely **(A)** high content of aromaticity, **(B)** binary alternating patterns of hydrophobic-hydrophilic residues and **(C)** surfactant-like, followed by **(D)** the most common supramolecular morphologies. **(E)** Molecular designs used for peptide-based virus mimetics based on capsid-like materials (tripodal and β-annulus designs) and peptide-DNA/RNA complexes. **(F)** AFM and TEM example images of fibrillar and spherical peptide nanostructures, adapted with permission from (Son et al., 2019). Copyright 2019 American Chemical Society.

In the context of molecular self-assembly, the composition and the physico-chemical properties of amino acid side chains dictate their behavior in different environments. In a hydrophilic environment, aromatic amino acids tend to aggregate due to hydrophobic interactions and π-π stacking, whereas polar and charged amino acids promote nanostructure formation through hydrogen bonds and electrostatic interactions, respectively. In addition, the position of a particular amino acid within the sequence, as well as the type of neighboring residues, affect the formation of supramolecular assemblies and their morphologies. Although it is possible to identify distinct sequence patterns with the tendency to form a particular nanostructure, it is challenging to attribute a supramolecular morphology based solely on the amino acid composition. Peptide sequences can self-assemble into a variety of shapes, including spheres, fibers, vesicles and tubes, with diameters in the 10–100 nm range and in the case of nanofibers, reaching micrometers in length (Gazit, 2007a; Zhao et al., 2008). In this section, we will focus on three main patterns used in the design of purely peptidic materials (Figures 1A–C): (i) high content of aromaticity, (ii) binary alternating hydrophobic-hydrophilic and (iii) surfactant-like.

### Peptides With High Content of Aromaticity

Peptides composed of aromatic amino acids preferentially self-assemble into nanofibers with high intermolecular β-sheet content. Short motifs such as FF, YY, and WW constitute fundamental building blocks for self-assembly, with diphenylalanine being the most widely studied one (Gazit, 2007b; Frederix et al., 2015; Tao et al., 2017). Depending on the combination of amino acids adjacent to the FF motif and its position within the sequence, various morphologies such as fibrous and plate-like assemblies for FFF, nanospheres for CFF, helical fibrils for PFF and heterogeneous nanostructures for FFV, VFF, and LFF, are observed (Reches and Gazit, 2004; Tamamis et al., 2009; Marchesan et al., 2012; Frederix et al., 2015; Bera et al., 2019). Examples of fiber forming peptides (Figure 1A) having longer sequences include FFKLVFF, GFFLG, GFFL, FFAGL, FFVLK, FFVDF, and WWWW (Kalafatovic et al., 2016; Diaferia et al., 2018; Son et al., 2019; Yang et al., 2020b). Moreover, tyrosine-rich sequences including YYAYY, YYACAYY, YFCFY, KYF, and KYY were found to assemble into nanosheets, nanocapsules or nanofibers (Frederix et al., 2015; Lee et al., 2019; Sloan-Dennison et al., 2021). Amyloid-like peptides find applications in plaque-associated neurodegenerative diseases research or as biosensors and nanocarriers (Gazit, 2007b; Al-Halifa et al., 2019).

### Binary Alternating Sequences of Hydrophobic-Hydrophilic Residues

Peptides with the binary-alternating patters rely on hydrogen bonds and/or electrostatic interactions for the formation of supramolecular assemblies. The first reported self-assembling peptide, EAK16-II (AEAEAKAKAEAEAKAK) is a repetitive segment derived from a natural yeast protein consisting of alternating hydrophobic and hydrophilic, positively, and negatively charged amino acids (Zhang et al., 1993; Zhang, 2017). It was shown that the disposition of amino acids within the sequence and the pH of the environment influence the supramolecular morphology of EAK16 (Hong et al., 2003). At neutral pH, EAK16-II formed fibrils and its analogue EAK16-IV (AEAEAEAEAKAKAKAK) formed globular assemblies, whereas both peptides showed fibrillar assemblies at conditions above or below the neutral. Other examples (Figure 1B) including RAD16-I (RADARADARADARADA), RAD16-II (RARADADARARADADA), KLDLKLDLKLDL, FKFEFKFE, FEFKFEFK, FEFEFKFK, VEVE, SFSFSF, RFRFRF, and NININI have been reported to self-assemble into fibers (Kisiday et al., 2002; Marini et al., 2002; Yokoi et al., 2005; Cui et al., 2009; Guilbaud et al., 2010; Mandal et al., 2014; Do et al., 2016; Gao et al., 2017; Pelin et al., 2020). In addition to linear sequences, cyclic peptides have been used as building blocks allowing for manipulation of the supramolecular morphology through monomer design (Mandal et al., 2013). Cyclic peptides having the [WR]_n_ structure, where n ∈ {3,4,5}, favor the formation of vesicle-type assemblies, unlike the linear designs with the binary alternating pattern, that preferentially assemble into fibrillar morphologies. The main applications of peptides classified in this category are related to their ability to form hydrogels. Such biomaterials can serve as scaffolds for tissue engineering, bioprinting, cell proliferation, regenerative medicine and drug delivery (Liu and Zhao, 2011; Levin et al., 2020; Gelain et al., 2021).

### Surfactant-Like Peptides

Surfactant-like peptides formed by combining aliphatic and charged segments have been also reported as self-assembly units. Their design is based on a hydrophobic tail composed of V, I, L, G, A or P followed by a charged head group containing K, D, R or E (Figure 1C). Examples include V_6_K, V_6_K_2_, V_6_D, V_6_D_2_, I_3_K, I_6_K_2,_ A_3_K, A_6_K, A_6_D, G_4_D_2_, G_6_D_2_, G_8_D_2_, A_12_R_2_, A_2_V_2_L_3_WE_2/7_, P_10_R_3_, etc. (Vauthey et al., 2002; van Hell et al., 2007; Yoon et al., 2008; Wang et al., 2009; Zhao, 2009; Xu et al., 2010; Hamley et al., 2013). These sequences can assemble into various morphologies comprising micelles, fibers, vesicles and tubes. The size and shape of the formed supramolecular assemblies depend on the type of amino acids used to constitute the amphiphile as well as the number of aliphatic and charged residues. In addition, factors such as temperature, solution pH and ionic strength affect the self-assembly process. The ability to form lipid bilayer-like assemblies makes them ideal for applications in immunotherapy, gene and drug delivery. Moreover, they can be used as protective envelopes for the delivery of enzymes and other biomolecules (Dasgupta and Das, 2019).

## Dimensional Control Through Co-Assembly

Compared to the unimolecular assemblies described above, supramolecular co-assemblies lead to the formation of nanostructures with increased chemical diversity and structural complexity that can resemble natural systems. Peptides can co-assemble in cooperative, orthogonal, disruptive or random manner (Makam and Gazit, 2018). It is possible to fine-tune the morphology and dimension of nanostructures, and consequently their chemical, mechanical and physical properties, by controlling the mixing ratio of the individual building blocks. For example, the co-assembly of FF and FFF can be tuned to obtain nanorods, spherical nanovesicles, hollow nanotubes and toroid-like nanostructures (Guo et al., 2016). The disruptive co-assembly of the FF motif with its capped version Boc-FF allowed for the precise control of the nanotube length from 12 to 8 µm by changing the mixing ratio from 20:1 to 5:1, respectively (Adler-Abramovich et al., 2016). The cooperative co-assembly of dendrimeric poly (lysine) hydrophilic heads with linear poly (leucine) hydrophobic tails allowed the morphology control of the formed peptidosomes by alternating the ratio of dendrimeric to linear component. When the ratio changed from 10:1 to 1:5, the size of the nanoparticles increased from 300 to 800 nm and their morphology changed from spherical to fusiform (Xu et al., 2012). The ability to achieve dimensional control constitutes a promising tool for the design of peptide supramolecular materials where specific morphologies or dimensions are required. However, the co-assembly of peptide-based nanomaterials has not been researched extensively and constitutes an opportunity to improve the future design of peptide materials (Sasselli et al., 2017).

## Viral Mimetics

Recently, the concept of mimicking viral capsids by creating their simplified versions through molecular self-assembly following the bottom-up strategy has emerged. Peptide self-assembly is a powerful tool to create biocompatible, tunable, low-cost supramolecular materials. It allows the conversion of chemically simple building blocks into a wide range of supramolecular architectures featuring modularity, functional diversity, adaptability and responsiveness to stimuli (Lampel, 2020). Peptides are versatile molecules for the design of virus mimetics as they can act as structural components as well as functional domains that favor selective binding, cell entry, endosomal escape or possess a specific activity (e.g., antimicrobial or catalytic). Short peptides offer the possibility to use minimal recognition modules for the design of functional materials and offer unique platforms for mimicking complex systems (Levin et al., 2020).

In this review, we distinguish the role of peptidic components used for the fabrication of virus mimetics into structural and functional modules (table 1). The structural modules are based on patterns that drive the formation of ordered supramolecular nanostructures having spherical or fibrillar morphologies (Figure 1D, F), dictated by the sequence, as described in section 2. In addition, trigonal cores (peptidic or organic), linkers (glycine or alkyl), cysteine residues serving as conjugation points, coiled coil, α-helical or β-annulus segments contribute to the design of structural modules. On the other hand, functional modules, related to the inherent biological signaling typical for peptides, are based on sequences with known activities such as cell penetration, integrin binding, DNA condensation and antimicrobial activity. Accordingly, peptide-based viral mimetic designs are divided into: i) capsid reconstruction strategies where structural modules contribute to the final supramolecular morphology and ii) simplified virus-like complexes where structural and functional peptidic modules are complexed with DNA or RNA fragments.

**TABLE 1:** Examples of peptide-based viral mimetic design strategies.

Strategy	Peptidic component / sequence	Role (structural (s) / functional (f))	Supramolecular Morphology	Development Stage	Ref.
**Capsid mimicking nanomaterials with C_3_ symmetry (trigonal or based on α-helical or β-sheet forming peptides)**					
Trigonal (trimesoyl) peptide conjugate	C^i^-FKFEFKFE^ii^	i) Conjugation to core molecule (s)	Nanospheres	Biophysical data	Matsuura et al. (2005)
	Ci-KTWTWTE^iii^	ii) β-sheet self-assembly unit (s)			Matsuura et al. (2011)
	(γE-C^i^-G)^iv^	iii) Tryptophane zipper based β-sheet self-assembly (s)			Matsuura et al. (2009)
		iv) self-assembly unit (s)			
Wheel (trimesoyl) peptide conjugate	(FKFE-C^i^-KFE)^ii^	i) Conjugation to core molecule (s)	Nanofibers	Biophysical data	Murasato et al. (2008)
		ii) β-sheet self-assembly unit (s)			
Trigonal (tertiary amine) dipeptide conjugate	WW	β-sheet self-assembly unit (s)	Nanospheres	Biophysical data	Ghosh et al., (2007)
	FF		Nanotubes		
Trigonal (ethyl benzene)-peptide conjugate	(γE-C^i^-G)^ii^	i) Conjugation to core molecule (s)	Nanospheres	Biophysical data	Matsuura et al., (2010a)
		ii) Self-assembly unit (s)			
Peptide triskelion (trilateral honeycomb symmetry)	_β_AKK^i^-(RRWTWE)3^ii,^^iii^	i) Trigonal core (s)	Nanocapsules	Cell assays (RNA delivery, antimicrobial activity)	Castelletto et al. (2016)
		ii) Tryptophane zipper based β-sheet self-assembly (s)			
		iii) Antimicrobial activity (f)			
Trigonal peptidic coiled coil heterodimers	K_β_AK_β_AK^i^-(KIAKLKQKIQKLKAKIAKLKQ)_3_^ii^	i) Trigonal core (s)	Nanospheres	Cell assays (RNA delivery, antimicrobial activity)	De Santis et al., (2017)
	C_β_AEISALEQEIASLEQEISALEQ^iii^	ii) Cationic, covalently bound antimicrobial component (s)			
		iii) Anionic component for heterodimer formation (s)			
β-annulus fragment from TBSV capsid	INHVGGTGGAIMAPVAVTRQLVGS^i^	i) β-annulus segment (s)	Hollow nanocapsules	Biophysical data	Matsuura et al. (2010b); Fujita and Matsuura (2017)
	INHVGGTGGAIMAPVAVTRQLVGG^i-^CGGGKIAALKKKNAALKQKIAALKQ^ii^	ii) Cationic component covalently bound to β-annulus (s)	Nanospheres		
	EIAALEKENAALEQEIAALEQ^iii^	iii) Anionic component for heterodimer formation (s)			
β-annulus fragment from SMV	GISMAPSAQGAM^i^-FKFE^ii^	i) β-annulus segment (s)	Nanospheres	Biophysical data	Matsuura et al., (2016)
		ii) β-sheet self-assembly unit (s)			
Tecto-dendrimeric design	C^i^-GG^ii^-EIARLEQEIARLEQEIARLEYEIARLE^iii^	i) Disulfide crosslinking (s)	Spherical particles	Cell assays (gene transfection)	Noble et al., (2016)
		ii) Glycine linker (s)			
		iii) α-helical conformation promoting sequence (s)			
**Multicomponent peptide-DNA complexes**					
Surfactant-like sequences	I_3_V_3_A_3_G_3_^i^-K3^ii^	i) β-sheet self-assembly unit (s)	Nanosheets for peptidic component	Cell assays (gene transfection)	Cao et al., (2018)
		ii) DNA condensing (f)	Heterogeneous morphologies *via* condensation with DNA		
Multicomponent, glucose-peptide conjugate	GSGSGS^i^-K_8_^ii^-GGSGGS^iii^-(WKWE)_3_WG^iv^	i) linker (s)	β-nanoribbons for peptidic component and for complexes with siRNA and dsDNA	Cells assays (siRNA transfection)	Lim et al., (2008)
		ii) siRNA binding site (f)			
		iii) linker (s)			
		iv) β-sheet self-assembly unit (s)			
Cocoon-like viral mimics based on β-sheet forming sequences (C6 = alkyl linker)	K_3_^i^-C_6_-WLVFFAQQ^ii^-G^iii^-SPD^iv^	i) cationic, DNA binding region (f)	Nanoribbons for peptidic component	Biophysical data	Ni and Chau, (2014)
		ii) amyloid / β-sheet segment (s)	Nanococoons *via* condensation with DNA		
	K_3_^i^-C_6_-X^ii^-G^iii^-SPD^iv^ where X ∈ {L_8_, L_6_, L_4_, A_8_, A_6_, (L_2_A_2_)_2_}	iii) glycine linker (s)	Nanofibers for peptidic component	Cell assays (gene transfection)	Ni and Chau, (2017)
		iv) hydrophilic segment (s)	Nanococoons *via* condensation with DNA for L_8_, L_6_, L_4_, (L_2_A_2_)_2_		
Tat–LK15 conjugate	(RKKRRQRRRGGG^i^-KLLKLLLKLLLKLLK^ii^)^iii^	i) Cell penetrating (f)	Peptide-DNA complex (morphology not determined)	Cells assays (gene transfection)	Saleh et al. (2010)
		ii) Membrane lytic, amphipathic (f)			
		iii) DNA binding (f)			
Multicomponent	K_6_^i^-GGFLG^ii^-FWRGENGRKTRSAYERMCNILKGK^iii^	i) DNA binding (f)ii) Enzyme cleavable (s/f)iii) Influenza-derived epitope (f)	Dimer formation through disulphide linkage for peptide component	Cells assays (gene transfection)	Haines et al. (2001)
			Spherical aggregates in presence of DNA		

Multicomponent, bola-amphiphile	RGD^i^-GPLGLAG^ii^-I_3_^iii^-G-R_8_^iv^	i) Integrin binding (f)	Nanospheres for peptidic component only	Cells assays (gene transfection)	Wang et al. (2020)
		ii) enzyme cleavable (hydrophobic) (s/f)	Rod-like or spherical shapes in presence of DNA		
		iii) structural (hydrophobic) (s)			
		iv) Cell penetrating, DNA binding (f)			
Bi-functional bola-amphiphile with hydrocarbon (C_12_) core	RGD^i^-C_12_-R_8_^ii^	i) Integrin binding (f)	Spherical nanoparticles *via* condensation with DNA	Cells assays (gene transfection)	Chen et al., (2013)
		ii) Cell penetrating, DNA binding (f)			

### Capsid-Like Nanomaterials

Viral capsids with icosahedral symmetry formed through the assembly of multiple protein subunits have inspired the design of artificial, peptide-based nanostructures for applications in gene delivery and cancer immunotherapy (Matsuura, 2018; Cai et al., 2020). In capsid reconstruction, it is important to maintain the n-fold rotational symmetry with n ∈ {3,5}. The C_3_ assembly can be achieved at the molecular level by designing trigonal conjugates or through folding-assembly pathways of peptides with helical conformations or β-annulus segments found on capsid-forming proteins (Figure 1E).

Trigonal designs induce the symmetry through the manipulation of the tripodal core (organic or peptidic) conjugated to peptidic structural modules that favor β-sheet-like self-assembly including the WTW tryptophane zipper and the FKFE-based binary alternating pattern. Examples are trymesoyl conjugates bearing three β-sheet-forming sequences (CFKFEFKFE or CKTWTWTE) attached through the C-terminal cysteine, that assemble into spherical morphologies (Matsuura et al., 2005, 2011). Similarly, a wheel-like trigonal design where the same core is conjugated to FKFECKFE through the central cysteine residue, self-assembled into fibers (Murasato et al., 2008). A clathrin triskelion-inspired conjugate, having a tris(2-aminoethyl)amine core linked to three aromatic di-tryptophan modules, self-assembled into nanospheres. In contrast, the conjugate containing the FF motif linked to the same core resulted in the formation of nanotubes, indicating that the morphology of the assemblies could be tuned through the dipeptide sequence (Ghosh et al., 2007). Furthermore, the choice of the core molecule can influence the properties of the obtained assemblies. For glutathione (_γ_ECG) attached to two different cores, the 1,3,5-tris(aminomethyl)-2,4,6-triethyl benzene showed improved conformational rigidity compared to the trimesoyl, giving rise to nanospheres with narrow size distribution (Matsuura et al., 2009; Matsuura et al., 2010a).

A purely peptidic triskelion, designed by conjugating each amino acid of the core sequence _β_AKK to the antimicrobial RRWTWE peptide containing the virus-derived tryptophane zipper, self-assembled into nanocapsules with dual function consisting of siRNA delivery and intrinsic antimicrobial activity (Castelletto et al., 2016). In this case, the RRWTWE sequence contains both the structural (β-sheet-forming) and functional (antimicrobial) modules. In another example, the core K_β_AK_β_AK sequence was conjugated to a positively charged antimicrobial (KIAKLKQKIQKLKAKIAKLKQ) peptide to form a trigonal conjugate, that upon addition of a complementary anionic sequence (C_β_AEISALEQEIASLEQEISALEQ), assembled in a coiled-coil hetero dimer. The resulting C_3_ subunit gave rise to capsid-like nanomaterials with antimicrobial activity (De Santis et al., 2017).

The reconstruction of capsid morphology based on the assembly of β-annulus peptide segments from Tomato bushy stunt virus (INHVGGTGGAIMAPVAVTRQLVG) and *Sesbania* mosaic virus (GISMAPSAQGAM) is able to maintain the C_3_ symmetry while allowing for introduction of surface modifications (Matsuura et al., 2010b; Matsuura et al., 2016). Among others, these include coating with gold nanoparticles to enhance the imaging efficiency (Matsuura et al., 2015) or with albumin to confer greater serum stability without eliciting immune response or toxicity (Matsuura and Honjo, 2019).

At the sequence level, the β-annulus segments can be modified with β-sheet promoting sequences (FKFE) to improve their assembly propensity into spherical morphologies (Matsuura et al., 2016). Moreover, with the intention of mimicking spike-bearing viruses such as Influenza and SARS-CoV-2, the β-annulus segment covalently linked to a cationic, coiled-coil-forming sequence at the C-terminus (CGGGKIAALKKKNAALKQKIAALKQ) gives rise to nanospheres. In the presence of a complementary anionic peptide (EIAALEKENAALEQEIAALEQ) and depending on the ratio of the cationic to anionic component, spherical (4:1) or fibrillar (1:1) assemblies with surface-exposed dimeric coiled coils are obtained (Fujita and Matsuura, 2017).

Another strategy is the use of a tecto-dendrimeric architecture as template to achieve C_3_ assembly into spherical particles for gene delivery. The design is based on structural coiled-coil subunits (CGG-EIARLEQEIARLEQEIARLEYEIARLE) configured into helical wheels, containing a GG spacer motif adjacent to a cysteine residue allowing for disulfide crosslinking (Noble et al., 2016).

### Multicomponent Peptide-DNA Complexes

Virus-mimicking nanostructures can be formed through the complexation of peptides with DNA or RNA (Figure 1E), simulating the co-assembly of capsid proteins with viral genomes. Predominantly positively charged peptides have the tendency to condense negatively charged gene fragments making the resulting virus mimicking nanostructures ideal candidates for gene delivery (Miyata et al., 2012). Compared to conventional, cytotoxic DNA condensation agents such as polyelectrolytes and lipidic surfactants, short peptides have higher biocompatibility and consequently lower toxicity. Moreover, their structure can easily be modified to obtain high affinity DNA binders (Wang et al., 2020). Furthermore, the condensation with the peptidic vector confers protection from DNases. Several peptide-DNA/RNA co-assemblies have been reported containing structural or functional modules or their combination resulting in multicomponent designs.

Peptide-DNA condensates composed of lysine modified surfactant-like, binary alternating or amyloid-like structural modules, have been reported. While the cationic region drives the binding to DNA or RNA through electrostatic attraction, peptide self-assembly and β-sheet formation takes place *via* hydrogen bonds and hydrophobic interactions. Surfactant-like sequences, obtained by varying the position of aliphatic amino acids (A, G, I, and L) as well as the position of the cationic (K_3_) region from N- to C- terminus, including cone-like (G_3_A_3_V_3_I_3_K_3_, K_3_I_3_V_3_A_3_G_3_), dumbbell-like (I_3_V_3_A_3_G_3_K_3_, K_3_G_3_A_3_V_3_I_3_) and irregular shaped sequences (V_3_G_3_I_3_A_3_K_3_, K_3_A_3_I_3_G_3_V_3_) gave rise to nanorods, nanosheets and nanofibrils, respectively. The I_3_V_3_A_3_G_3_K_3_ was the most efficient one in inducing DNA condensation showing high content of ordered domains (Cao et al., 2018). This example shows that the supramolecular morphology and content of ordered domains could be fine-tuned through sequence engineering. Furthermore, a glucose-peptide conjugate [Glucose-GSGSGS-K_8_-GGSGGS-(WKWE)_3_WG] containing a functional, cationic segment (K_8_) for siRNA binding positioned between two linkers (GSGSGS and GGSGGS) and a binary alternating structural motif (WKWE)_3_, assembled into bilayered β-nanoribbons. The carbohydrate ligand exhibited the dual function of maintaining the β-nanoribbons neutrally charged while enhancing the cell binding through glucose transporters (Lim et al., 2008). Therefore, this design offers the formation of a controllable filamentous morphology able to bind RNA while presenting surface functionalization that yields high transfection efficiency.

Another example is the design of the cocoon-like virus mimetics based on a sequence (K_3_-C_6_-WLVFFAQQGSPD) containing the cationic, DNA binding region (K_3_) at the N-terminus, followed by the alkyl linker (C_6_) and three structural components, namely, the amyloid-like motif (LVFFA), the glycine linker and the hydrophilic (SPD) region (Ni and Chau, 2014). The β-sheet forming segment can be modified from amyloid to aliphatic (L_8_, L_6_, L_4,_ and L_2_A_2_L_2_A_2_) while maintaining the self-assembly propensity of the whole sequence. The peptides alone self-assemble into fibrillar aggregates, while their interaction with DNA in various ratios induces condensation into nanococoons (Ni and Chau, 2017).

Cell penetrating peptides including the arginine-rich, R_8_ and the HIV-1 derived, Tat (RKKRRQRRRGGG) constitute the main functional modules used for the design of DNA condensates (Kalafatovic and Giralt, 2017). The covalent conjugation of Tat to the amphipathic LK15 sequence (KLLKLLLKLLLKLLK) resulted in improved cellular uptake and transfection efficiency, compared to Tat or LK15 alone (Saleh et al., 2010). CL22 (K_6_-GGFLG-FWRGENGRKTRSAYERMCNILKGK) is an example of purely peptidic design containing an enzyme cleavable segment adjacent to the DNA binding region at the N-terminus and the Influenza nucleoprotein-derived sequence at the C-terminus. It assembles into spherical aggregates in the presence of DNA and attains maximum gene transfection efficiency upon spontaneous dimerization through the disulfide bond between cysteines at the C-terminus (Haines et al., 2001). Bola amphiphiles, composed of a central hydrophobic segment flanked by two hydrophilic ones, have the ability to self-assemble into fibrillar or spherical nanostructures depending on the sequence design (Chen et al., 2013). Examples are the purely peptidic RGD-GPLGLAG-I_3_-G-R_8_ (Wang et al. 2020) and the fatty acid containing RGD-C_12_-R_8_ (Chen et al., 2013) that accommodate both functional and structural motifs, where RGD is crucial for integrin-binding and R_8_ for cell penetration. Additionally, the PLGLA sequence serves as an enzyme-cleavable segment, while I_3_ confers hydrophobicity. The main drawback of peptide-DNA/RNA co-assemblies, mainly based on functional modules, is that oppositely charged polyions often form heterogeneous aggregates. The challenges resulting from the lack of control over their morphology, degree of order and size, often hamper the efficiency of gene transfection or delivery.

The DNA fragment length and composition can affect the formation of peptide-DNA complexes but also their morphology. The mechanism of formation depends on the peptides’ intrinsic ability to self-assemble. Self-assembling peptide sequences condense the DNA by reorganizing to a final morphology that is often different from the one formed by the peptide alone. On the other hand, predominantly cationic and/or cell-penetrating peptides, unable to self-assemble, tend to form irregular aggregates in the presence of DNA. Moreover, the size of the complex can be controlled by varying the length of the DNA fragment. For example, the I_3_V_3_A_3_G_3_K_3_–DNA complex size decreased from 122 to 85 nm by shortening the DNA fragment from 2000 to 300 bp (base pairs). Even though most examples use λ-DNA (∼4.8 kbp), shorter DNA fragments (2000—300 bp) were explored with the intention to improve the DNA delivery efficiency (Cao et al., 2018).

However, the key factor influencing the morphology of peptide-DNA complexes is the R + / − ratio of the positively charged peptide residues to the negatively charged DNA fragments. A stable peptide-DNA complex is formed when all the negative charges are successfully neutralized. For example, RGD-GPLGLAG-I_3_-G-R_8_ that self-assembles into spheres, upon the interaction with DNA and depending on the R + / - values forms thread-like (R + / - = 0.5) complexes or highly condensed rod-like or spherical (R + / - = 3) nanostructures (Wang et al., 2020). In another example, the R + / - of 10 is the minimum requirement for DNA condensation with K_3_C_6_SPD, where the peptide alone self-assembles into nanoribbons. However, upon DNA addition, the electrostatic interactions drive the self-assembly into amorphous aggregates (R + / - = 5), or agglomerations with small striped nanococoons (R + / - = 10). The R + / - = 20 presents the optimal ratio for nanococoon formation, while at R + / - of 25 and 50 both nanococoons and filamentous nanoribbons are formed (Ni and Chau, 2014).

## Future Perspectives

The idea of exploiting known principles of peptide self-assembly to obtain spherical or fibrillar nanostructures by including important features such as cell penetration, antimicrobial activity or viral transfection is conceptually attractive. Such systems are promising as they can be easily engineered and modified to include specific sequences found on the receptor binding domains of spike proteins. In addition, they can be designed as vehicles able to deliver cargo into cells. So far, morphology rather than functionality has been mimicked and it constitutes an advantage from the point of view of easy production compared to VLPs. A step towards functionality of peptide materials is their ability to enhance viral transfection by increasing the β-sheet content of supramolecular nanostructures (Sieste et al., 2021). However, efforts are needed to achieve controllable and complex functions such as self-replication and catalysis in the future. Although largely unexplored for clinical use, because of the multiscale and multiparameter optimization challenges of supramolecular nanostructures (Sieste et al., 2021), we envision that peptides have great potential in becoming future nanotechnological solutions in covid-19 therapy and diagnostics.

The intention of this review is to emphasize the increasing importance of peptide self-assembly in the design and fabrication of minimalistic, synthetic models applicable to a variety of viral infections. We expect that future research in this field will deliver simple and cost-effective viral mimetics composed of peptide modules found on the surface of specific viruses, rationally designed to assemble into multivalent and multifunctional nanostructures able to selectively bind receptors of interest, penetrate cells and carry cargos. In addition to mimicking the viral morphology, such systems would partly resemble basic functionality through the display of known functional modules and their combinations aiming for possible synergistic effects. Such an approach could lead to the development of efficient and safe platforms to study viral infections without the need of complicated genetic manipulations. Moreover, the developed models will provide screening platforms that can be rationally designed, allowing for rapid discovery of potential inhibitors or surface protein binders. Therefore, they could be used as safe alternatives for antiviral drug discovery or as vehicles for mRNA vaccines.
